# Experimental Therapy of HER2-Expressing Xenografts Using the Second-Generation HER2-Targeting Affibody Molecule ^188^Re-ZHER2:41071

**DOI:** 10.3390/pharmaceutics14051092

**Published:** 2022-05-20

**Authors:** Yongsheng Liu, Anzhelika Vorobyeva, Anna Orlova, Mark W. Konijnenberg, Tianqi Xu, Olga Bragina, Annika Loftenius, Erica Rosander, Fredrik Y. Frejd, Vladimir Tolmachev

**Affiliations:** 1 Department of Immunology, Genetics and Pathology, Uppsala University, 751 85 Uppsala, Sweden; yongsheng.liu@igp.uu.se (Y.L.); anzhelika.vorobyeva@igp.uu.se (A.V.); tianqi.xu@igp.uu.se (T.X.); fredrik.frejd@affibody.se (F.Y.F.); 2 Department of Medicinal Chemistry, Uppsala University, 751 83 Uppsala, Sweden; anna.orlova@ilk.uu.se; 3 Department of Radiology and Nuclear Medicine, Erasmus MC, 3000 CA Rotterdam, The Netherlands; m.konijnenberg@erasmusmc.nl; 4 Research Centrum for Oncotheranostics, Research School of Chemistry and Applied Biomedical Sciences, Tomsk Polytechnic University, 634050 Tomsk, Russia; bragina_od@mail.ru; 5 Affibody AB, 171 65 Solna, Sweden; annika.loftenius@affibody.se (A.L.); erica.rosander@affibody.se (E.R.)

**Keywords:** HER2, radionuclide therapy, affibody molecule, rhenium-188, second-generation scaffold

## Abstract

HER2-targeted radionuclide therapy might be helpful for the treatment of breast, gastric, and ovarian cancers which have developed resistance to antibody and antibody-drug conjugate-based therapies despite preserved high HER2-expression. Affibody molecules are small targeting proteins based on a non-immunoglobulin scaffold. The goal of this study was to test in an animal model a hypothesis that the second-generation HER2-targeting Affibody molecule ^188^Re-ZHER2:41071 might be useful for treatment of HER2-expressing malignant tumors. ZHER2:41071 was efficiently labeled with a beta-emitting radionuclide rhenium-188 (^188^Re). ^188^Re-ZHER2:41071 demonstrated preserved specificity and high affinity (K_D_ = 5 ± 3 pM) of binding to HER2-expressing cells. In vivo studies demonstrated rapid washout of ^188^Re from kidneys. The uptake in HER2-expressing SKOV-3 xenografts was HER2-specific and significantly exceeded the renal uptake 4 h after injection and later. The median survival of mice, which were treated by three injections of 16 MBq ^188^Re-ZHER2:41071 was 68 days, which was significantly longer (<0.0001 in the log-rank Mantel-Cox test) than survival of mice in the control groups treated with vehicle (29 days) or unlabeled ZHER2:41071 (27.5 days). In conclusion, the experimental radionuclide therapy using ^188^Re-ZHER2:41071 enabled enhancement of survival of mice with human tumors without toxicity to the kidneys, which is the critical organ.

## 1. Introduction

Overexpression of human epidermal growth factor receptor 2 (HER2) in breast, gastroesophageal and ovarian cancers is utilized for targeted therapy. Anti-HER2 monoclonal antibodies (Mab), trastuzumab and pertuzumab, are used for treatment of HER2-expressing tumors and prolong survival of patients with HER2-positive breast and gastric cancers [1,2]. However, a substantial fraction of tumors develops resistance to trastuzumab during therapy despite preserving a high level of HER2 expression [3]. Anti-cancer action of Mabs might be further extended by a conjugation of cytotoxic drug to tumor-targeting antibodies [4]. The use of antibody-drug conjugate trastuzumab-DM1 (T-DM1) provided a high response rate in pre-treated patients whose tumors expressed more than median HER2 levels [5,6]. However, a variety of resistance mechanisms result in ultimate progression of HER2-expressing tumors treated with T-DM1 [7].

Radionuclides, which emit beta- or alpha-particles, are considered as cytotoxic payloads that might be a possible alternative or addition to cytotoxic drugs. It is expected that radionuclide therapy would not be affected by multidrug resistance. Antibody-mediated radionuclide targeted therapy has demonstrated efficacy against radiosensitive hematologic malignancies. However, the use of labeled full-length antibodies for radionuclide therapy of solid tumors failed to demonstrate clinical efficacy [8]. One of the obstacles is the fact that the size of Mabs is relatively large (150 kDa). They remain in circulation for a long time, irradiating the radiosensitive bone marrow. In addition, radiolabeled Mabs do not efficiently penetrate in the tumor tissue. Both these factors limit the delivery of sufficient absorbed dose to tumors without delivering unacceptably high dose to critical normal organs. A promising approach to overcome these limitations is the development of smaller tumor-targeting agents with high affinity such as engineered scaffold proteins (ESPs).

Affibody^®^ molecules are one of possible ESPs for in vivo radionuclide targeting [9,10]. These small proteins are derived from a 58-amino-acid (7 kDa) triple-helix bundle scaffold, which is capable of refolding after thermal or chemical denaturing. Molecular display techniques provide selection of Affibody^®^ molecules binding to several cancer-relating molecular targets with high specificity and high affinity [11]. Earlier, an Affibody^®^ molecule, ZHER2:342, which binds to HER2 with picomolar affinity (dissociation constant at equilibrium, K_D_, ~22 pM), demonstrated capability to target HER2-expressing xenografts in mice [12]. Derivatives of this Affibody^®^ molecule, which were labeled with ^111^In and ^68^Ga, have shown an excellent visualization of HER2-expression in breast cancer metastases during clinical trials [13,14]. However, a renal excretion of Affibody molecules is followed by nearly complete reabsorption of the protein in proximal tubuli. In the case of the use of a residualizing radiometal labels, this results in a strong activity retention in kidneys [15] leading to unacceptably high renal absorbed doses. Methods, that are commonly used for blocking of renal reabsorption of radiolabeled peptides, such as pre- or co-injection of cationic amino acids or Gelofusine, have no substantial effect in the case of Affibody^®^ molecules [16]. A possible solution was indicated by the fact that anti-HER2 Affibody^®^ molecules are slowly internalized by cancer cells [17]. This feature enables the use of non-residualizing labels since the presence of radionuclide in tumors is more dependent on a strong binding of a radiolabeled Affibody^®^ to a target on cellular membrane than on intracellular retention of radiometabolites in this case. On the opposite, the internalization of Affibody^®^ molecules in kidneys is rapid, which leads to a prompt leakage of a non-residualizing label from this organ [12,18]. Therefore, the tumor uptake appreciably exceeds the renal uptake a few hours after injection of an Affibody^®^ molecule with a non-residualizing label, which potentially enables a radionuclide therapy.

^99m^Tc-labeled anti-HER2 Affibody^®^ molecules containing C-terminal -GGGC chelator, ZHER2:V2, demonstrated higher uptake in tumors than in kidneys due to the non-residualizing properties of the ^99m^Tc-GGGC label [19]. The development of second-generation of Affibody^®^ molecules increased their thermal and chemical stability, enhanced hydrophilicity of their surface and enabled their facile chemical synthesis [20]. The profound re-engineering of the scaffold might cause a loss of site-specificity of the labeling with ^99m^Tc. However, the second- generation anti-HER2 Affibody^®^ molecule ^99m^Tc-ZHER2:41071 with the C-terminal –GGGC chelator showed the same low renal retention of activity as the ^99m^Tc-ZHER2:V2 [21].

Rhenium is a chemical analog to technetium. Two rhenium beta-emitting radioisotopes, rhenium-186 (E_βmax_ = 1.071 MeV, T_1/2_ = 3.7 d) and rhenium-188 (E_βmax_ = 2.1 MeV, T_1/2_ = 17 h) are suitable for radionuclide therapy [22]. ^188^Re is produced by the ^188^W/^188^Re generator. This offers an advantage of high specific activity of the radionuclide and its convenient availability in hospitals. High energy of β-particles, which are emitted by ^188^Re, makes it suitable for eradication of large metastases, which are expected in the case of therapy-refractory tumors. Low-abundance (15%) γ-radiation with low energy (155 keV) enables quantitative in vivo imaging of the biodistribution of ^188^Re-labeled conjugates in patients for dosimetry. Due to chemical similarity between ^99m^Tc and ^188^Re, the chelators suitable for ^99m^Tc are usually suitable also for ^188^Re, and biodistribution of compounds labeled with these nuclides is similar [23]. This was a rationale for development of ^188^Re-labeled ZHER2:41071 as a therapeutic agent for treatment of HER2-expressing malignant tumors.

The goal of this study was to test in an animal model the hypothesis that ^188^Re-ZHER2:41071 could be useful for treatment of HER2-expressing malignant tumors. For this purpose, specificity and affinity of ^188^Re-ZHER2:41071 binding to HER2-expressing cells in vitro were evaluated, its biodistribution in mice bearing HER2-expressin SKOV-3 xenografts was measured, and finally an experimental therapy study was performed.

## 2. Materials and Methods

### 2.1. General

Gluconic acid sodium salt, ethylenediaminetetraacetic acid (EDTA) and tin(II) chloride dihydrate were purchased from Sigma-Aldrich Sweden AB (Stockholm, Sweden). Buffers used for labeling were prepared using Milli-Q water. A ^188^W/^188^Re generator was purchased from OncoBeta GmbH (Garching bei München, Germany). The radioactivity was measured using an automated gamma-spectrometer with a NaI (TI) detector (2480 Wizard, PerkinElmer, Waltham, MA, USA). The activity distribution along the instant thin-layer chromatography (iTLC) strips was measured with a Storage Phosphor System (CR-35 BIO Plus, Elysia-Raytest, Bietigheim-Bissingen, Germany) and analyzed with AIDA Image Analysis software (Elysia-Raytest, Bietigheim-Bissingen, Germany). The Affibody^®^ molecule ZHER2:41071 was produced as described earlier [21]. The control Affibody molecule ZHER2:2395 with the original scaffold and KVDC chelator at C-terminus was produced as described earlier [24].

In vitro cell studies were performed using the HER2-expressing ovarian cancer SKOV3 and breast cancer SKBR3 cells. For preparation of HER2-negative xenografts, Ramos lymphoma cells were used. All cell lines obtained from the American Type Culture Collection (ATCC, Manassas, VA, USA). Cells were cultured in Roswell Park Memorial Institute (RPMI) 1640 medium (Sigma-Aldrich) supplemented with 10% fetal calf serum, 2 mM L-glutamine, 100 IU/mL penicillin and 100 mg/mL streptomycin. Data on in vitro studies and biodistribution were analyzed by unpaired 2-tailed t-test and ANOVA using GraphPad Prism (version 9.3.1 for Windows; GraphPad Software) to determine significant differences (*p* < 0.05).

### 2.2. Labeling Chemistry

^188^Re is obtained as perrhenate by elution of a ^188^W/^188^Re generator with 4 mL sterile 0.9% sodium chloride (both from OncoBeta GmbH, Germany). Radiolabeling is performed in two variants: with a low eluate volume (1500 µL or less) and with a high eluate volume (4000 µL).

*Labeling with a low eluate volume* was performed by adding the contents of one freeze-dried kit containing 1 mg tin(II) chloride dihydrate, 5 mg gluconic acid sodium salt and 100 µg EDTANa_4_, dissolved in 1.25 M sodium acetate, pH 4.2, to 100 µg of ZHER2:41071 to a total volume of 100 µL. To the reaction solution, up to 1000 µL (300 MBq) of a ^188^Re-containing generator eluate is added. The mixture is incubated at 70–90 °C for 60 min and then cooled at room temperature for 5 min. Purification of the labeled ^188^Re-ZHER2:41071 from components of the labeling solution was performed using a size-exclusion NAP-5 column, pre-equilibrated with a solution of 10 µg/mL tin(II) chloride dihydrate in 0.9% sodium chloride. The column was eluted with the same solution. The same protocol was also used for labelling of the control Affibody molecule ZHER2:2395.

*Labeling with a high eluate volume* was performed by adding the contents of one freeze-dried kit containing 2 mg tin(II) chloride dihydrate, 50 mg gluconic acid sodium salt and 200 µg EDTANa_4_, dissolved in 1.25 M sodium acetate, pH 4.2, to 200 µg of ZHER2:41071 to a total volume of 100 µL. To the reaction solution, 4000 µL of a ^188^Re-containing generator eluate is added. An equivalent of 440 µg ascorbic acid (2 mg/mL in 1.25 M sodium acetate buffer, pH 4.2) is added to the reaction vial and the mixture is incubated at 70 °C for 60 min and then cooled at room temperature for 5 min. Thereafter, the total amount of ascorbic acid in the reaction vial is adjusted to 1 mg using a solution of 5 mg/mL ascorbic acid in PBS. Purification was performed using Oasis HLB 1 cc Vac Cartridge (Waters). The cartridge was activated by passing 5 mL 50% ethanol in water and de-activated by passing 5 mL water. The reaction mixture was passed through the cartridge, followed by 20 mL water. The cartridge was additionally washed with 500 µL 25% ethanol in water and the purified ^188^Re-ZHER2:41071 was eluted with 1 mL 50% ethanol in water. For the further use, ^188^Re-ZHER2:41071 was diluted with water to final ethanol concentration of 5–10%.

Radiochemical yield and purity of Affibody^®^ molecules were analyzed using instant thin layer chromatography (iTLC-SG) (Agilent Technologies, Santa Clara, CA, USA) developed with PBS (^188^Re-ZHER2:41071: Rf = 0.0, other forms of ^188^Re: Rf = 1.0). The rhenium colloid amount in the product was measured using pyridine: acetic acid: water (5:3:1.5) as the mobile phase (^188^Re colloid: Rf = 0.0, other forms of ^188^Re and ^188^Re-ZHER2:41071: Rf = 1.0).

To validate iTLC, radio-HPLC of ^188^Re-ZHER2:41071 was performed. Elite LaChrom system (Hitachi, VWR, Darmstadt, Germany) consisting of an L-2130 pump, a UV detector (L-2400), and a radiation flow detector (Bioscan, Washington, DC, USA) coupled in series was used. The analysis was performed using an analytical column (Phenomenex, Aschaffenburg, Germany; Luna^®^ 5 µm C18, 100 Å; 4.6 × 150 mm). HPLC conditions were as follows: Solvent A = 10 mM TFA/H_2_O; Solvent B = 10 mM TFA/acetonitrile; gradient elution: 0–25 min at 5 to 70% B, 25–28 min at 70 to 95% B, 29–30 min at 5% B; and flow rate was 1.0 mL/min; UV-detection at 214 nm).

### 2.3. In Vitro Studies

Binding specificity of ^188^Re-ZHER2:41071 to HER2-expressing cells was evaluated in a saturation experiment using SKOV3 and SKBR3 cells. Experiments were performed in triplicate. Cells were seeded in 6-well plates (~1 × 10^6^ cells/well) the day before the experiment. For blocking, a 500-fold excess of non-labeled ZHER2:41071 was added to the cells in control wells 15 min before adding labeled ^188^Re-ZHER2:41071 to saturate the receptors. Thereafter, the cells were incubated with a 2 nM (molar activity approximately 4 GBq/µmol) solution of ^188^Re-ZHER2:41071 for 60 min at 37 °C. After incubation, the media was collected, the cells were washed with 1 mL PBS, and the fractions were pooled. Thereafter, the cells were detached by incubation with trypsin-EDTA solution. The radioactivity in cells and media was measured to calculate the percentage of cell-bound radioactivity.

The affinity of ^188^Re-ZHER2:41071 and ^188^Re-ZHER2:2395 to SKOV-3 cells was measured by LigandTracer (Ridgeview, Instruments AB, Vänge, Sweden). Briefly, SKOV-3 cells were seeded on a local area of a cell culture dish (NunclonTM, Size 100620, NUNC A/S, Roskilde, Denmark). ^188^Re-ZHER2:41071 (concentration 0.1 and 0.3 nM, molar activity approximately 4 GBq/µmol) was added to the cells. The association and dissociation kinetics was measured in real time as described in [25]. The data were analyzed by the InteractionMap software (Ridgeview Diagnostics AB, Uppsala, Sweden) as described by Altschuh et al. [26] to calculate association rate, dissociation rate and dissociation constant at equilibrium (K_D_).

Cellular processing of ^188^Re-ZHER2:41071 was studied in HER2-expressing SKOV-3 and SKBR-3 cells using a modified acid wash method [17]. ^188^Re-labeled ZHER2:2395 Affibody molecule with a residualizing ^99m^Tc-KVDC label was used as a comparator. Cells (7 × 10^5^ cells/dish) were incubated with 2 nM solution (molar activity approximately 4 GBq/µmol) of ^188^Re labeled ZHER2:41071 at 37 °C. At 1, 2, 4, 8 and 24 h after the incubation start, a group of three dishes was removed from the incubator and the medium was collected. The cells were then washed two times with PBS and treated with 0.2 M glycine buffer containing 4 M urea, pH 2.0, for 5 min on ice. The glycine buffer was collected and the cells were washed with additional 1 mL glycine buffer. The acidic fractions were pooled, their activity was measured and considered as membrane-bound activity. Thereafter, cells were incubated with 1 mL of 1 M NaOH at 37 °C for 20 min and collected with additional 1 mL NaOH. Their measured activity was considered as an internalized activity.

### 2.4. In Vivo Studies

The animal experiments were performed in accordance with national legislation on laboratory animal protection, and the study was approved by the local Ethics Committee for Animal Research in Uppsala.

Four mice per data point were used in biodistribution experiments. Mice were euthanized at pre-determined time points by an intraperitoneal injection of anesthesia, Ketalar-Rompun solution (Ketalar: 10 mg/mL, Rompun: 1 mg/mL). Organs of interest were excised, weighed and their activity was measured. The tissue uptake values were calculated as the percentage of injected dose per gram of the sample (%ID/g).

Initial biodistribution studies were performed in female Naval Medical Research Institute (NMRI) mice. To evaluate if radioperrhenate is generated during metabolism of ^188^Re-ZHER2:41071, an effect of in vivo blocking of Na/I symporter was evaluated. Two groups of mice were used to evaluate the effect of blocking of Na/I-symporter on biodistribution of activity. For one group of mice, 5% sodium iodide was added to their drinking water 24 h before the experiment. The average animal weight was 28.8 ± 2.9 g at the time of the experiment. Mice were injected intravenously with 5 µg (210 kBq) ^188^Re-ZHER2:41071 per mouse in 100 µL 0.9% sterile saline. The biodistribution was measured 4 h after injection.

To obtain the data for estimation of dosimetry in humans, the biodistribution in normal NMRI mice was measured 0.5, 1, 4, 24 and 48 h after injection of 5 µg (210 kBq) ^188^Re-ZHER2:41071 per mouse in 100 µL 0.9% sterile saline. Based on the results of the previous experiments, mice were given drinking water containing 5% sodium iodine. The average animal weight was 29.6 ± 3.4 g at the time of the experiment. The blood, heart, salivary gland, lung, liver, spleen, pancreas, kidney, muscle, bone, brain, stomach, small intestine, large intestine, and the remaining carcass were collected. The tissue uptake values were calculated as the percentage of injected dose per gram of the sample (%ID/g), while the stomach, intestines, and carcass uptake, were calculated as the percentage of injected dose (%ID) per whole sample.

For assessment of dosimetry in humans, the murine biodistribution data were upscaled using the “percent kg/g method” according to Equation (1).

(%IA/organ)_human_ = [(%IA/g)_animal_ × (kg_TBweight_)_animal_ × (g_organ_/(kg_TBweight_)_human_] (1)

The organ weight from reference adult female (ICRP publication 23) phantom were used for upscaling. The uptake value was fitted by an exponential function and areas under curves were calculated to determine residence times. OLINDA/EXM 1.0 software (Vanderbilt University, Nashville, TN, USA) was used to estimate absorbed doses.

Biodistribution of ^188^Re-ZHER2:41071 in tumor bearing mice was measured in female BALB/C nu/nu mice. To establish the HER2-positive and HER2-negative xenografts, approximately 10^7^ SKOV-3 cells or Ramos cells, respectively, were subcutaneously implanted in the hind legs of mice. The average animal weight was 18.3 ± 1.2 g at the time of the experiment. The average SKOV-3 tumor weight was 0.16 ± 0.12 g. The average Ramos tumor weight was 1.19 ± 0.62 g. Drinking water was supplemented with 5% sodium iodide 24 h before injection of ^188^Re-ZHER2:41071. Mice were injected intravenously with 10 µg (270 kBq) ^188^Re-ZHER2:41071 per mouse in 100 µL 0.9% sterile saline. At 1, 4, 8, 24, and 48 h after injection, one group of mice with SKOV-3 was euthanized and the biodistribution was measured. To test in vivo specificity, biodistribution and tumor uptake was measured 4 h after injection in one group of mice with Ramos xenografts.

In vivo imaging was performed to confirm the biodistribution data. Two mice with SKOV-3 xenografts and one mouse with Ramos xenografts were injected with 4 MBq (10 µg) of ^188^Re-ZHER2:41071. The imaging was performed 1 h and 4 h p.i. for mice bearing SKOV-3 xenografts and 4 h for mice bearing Ramos xenografts using nanoScan SPECT/CT (Mediso Medical Imaging Systems, Budapest, Hungary). The data were reconstructed using Tera-Tomo™ 3D SPECT Software.

To select the optimal injected activity in the planned experimental therapy, the dosimetry of ^188^Re-ZHER2:41071 was evaluated. The biodistribution data were used to determine the time-activity curves of uptake in organs and tumor. Exponential curves were fitted to the data using GraphPad Prism version 9.3.1 (GraphPad Software, San Diego, CA, USA). Time-integrated activity concentration coefficients (TIACC) were determined by integration over time of the exponential fits folded with the ^188^Re exponential decay function. Absorbed doses to the organs were determined by using the ^188^Re S-values for the 25 g RADAR the realistic mouse model [27]. Multiplication of the TIACC with the mouse model organ mass and with the S-value yields the absorbed dose per injected activity. Cross organ doses were also determined, including the cross dose from activity in the tumor by taking the testes as source organ. The absorbed dose to the tumor itself was determined with the S-value for a 0.1 mL sphere of lymphatic tissue: 2257 mGy/MBq.h, obtained with the IDAC-2.1 software [28].

The tumor response to this radiation exposure was based on the in-vivo survival model based on the linear quadratic (LQ) model and exponential tumor growth [29]. The surviving number of tumor cells *S*(*t*) after a dose *D* at time t after injection is expressed by:

(2) $$S\left(t\right)=e^{\gamma t}e^{-\alpha D-G\left(t\right)\beta D^{2}}$$

with γ the normal growth constant for the tumor and *G*(*t*) the Lea-Catcheside time factor:

(3) $$G\left(t\right)=\frac{\lambda_{e}}{\lambda_{e}+\mu }\left\{\frac{1-2\frac{\lambda_{e}}{\lambda_{e}-\mu }e^{-\left(\lambda_{e}+\mu \right)t}+\frac{\lambda_{e}+\mu }{\lambda_{e}-\mu }e^{-2\lambda_{e}t}}{{\left(1-e^{-\lambda_{e}t}\right)}^{2}}\right\}$$

where *λ_e_* is the effective decay constant and *μ* is the sublethal DNA damage repair constant which was taken to be 0.462 h^−1^, corresponding to a repair half-life of 1.5 h. The LQ model radiation sensitivity parameters α and β for SKOV-3 tumors were derived from clonogenic survival assay data after external beam exposure [30] with the following results: α = 0.043 Gy^−1^ and β = 0.048 Gy^−2^ (α/β = 0.90 Gy). The tumor volume is supposed to follow the total number of tumor cells over time. A rapid tumor volume doubling time of eight days was assumed, hence γ = 0.0036 h^−1^. Tumor control probability (TCP) was calculated on basis of the Poisson statistics for the surviving number of cells:

(4) $$TCP\left(t\right)=e^{-N_{0}S\left(t\right)}$$

The initial clonogenic cell density was set at 10^9^ cells/g, leading to *N*_0_ = 1.0 × 10^8^ clonogenic cells in a 100 mg tumor. The cell survival and *TCP* was evaluated for various dosing schemes at multiple therapy cycles and intervals between.

### 2.5. Experimental Radionuclide Therapy

To evaluate the therapeutic efficacy of ^188^Re-ZHER2:41071, thirty female BALB/C nu/nu mice were subcutaneously implanted on the abdomen with 10^7^ SKOV-3 cells. One week after the implantation, the mice were randomly distributed to three groups, A–C (10 mice per group). In group A (treatment with radiolabeled Affibody^®^ molecule), mice were injected with 5 µg (16 MBq) of ^188^Re-ZHER2:41071 in 150 µL of 10% ethanol in 0.9% saline. In group B (treatment with non-labeled Affibody molecule), mice were injected with 5 µg of ZHER2:41071 in 150 µL of 10% ethanol in 0.9% saline. In group C (vehicle-treated control), mice were injected with 150 µL of 10% ethanol in 0.9% saline. Three injections separated by 48 h were performed intravenously. The tumor volumes at the start of the treatment (day 0) were 101 ± 28, 85 ± 42, and 86 ± 37 mm^3^ for mice in group A, B, and C, respectively. Throughout the experiment, tumor volumes and body weights were measured twice per week. The largest longitudinal (length) and transverse (width) diameter were measured by the caliper and the tumor volume (V) was calculated by the formula: V = 1/2 × (length × width^2^). Mice were euthanized when the tumor volume exceeded 1000 mm^3^, or when bleeding ulcerations were observed, or when overall weight loss was over 15% or the weight loss was more than 10% within one week. According to the ethical permit, the study was terminated, and all mice were euthanized 90 days after the first injection. After that, tumors, kidneys, and livers were collected, fixed with formalin, and embedded in paraffin. The pathology examination was performed by an experienced animal pathologist at BioVet AB veterinary medicine lab (Sollentuna, Sweden). The samples were stained with hematoxylin and eosin and examined for histopathologic changes.

For imaging HER2 expression during experimental therapy, SPECT/CT scans of mice bearing SKOV3 xenografts were performed using nanoScan SPECT/CT (Mediso Medical Imaging Systems, Budapest, Hungary). Two mice from each group were injected with ^99m^Tc-labeled ZHER2:41071 [21] Affibody^®^ molecule (5 μg, 16 MBq) and imaging was performed at 4 h p.i.

Data were analyzed by an unpaired, two-tailed t-test or one-way ANOVA with Bonferroni correction for multiple comparisons using GraphPad Prism (version 8.0; GraphPad Software, Inc., La Jolla, CA, USA). A *p*-value < 0.05 was considered a statistically significant difference.

## 3. Results

### 3.1. Labeling Chemistry

A labeling of ZHER2:41071 with a low eluate volume provided a radiochemical yield of more than 98% (*n* = 8). The iTLC data were confirmed with radio-HPLC (Figure S1). A labeling of ZHER2:2395 using the same protocol resulted in radiochemical yields exceeding 95%. The radiochemical purity of ^188^Re-ZHER2:2395 after size-exclusion chromatography purification was over 99% A labeling of ZHER2:41071 with a high rhenium 188-eluate volume provided the radiochemical yield of 85 ± 10% and a radiocolloid content of 0.2 ± 0.1% (*n* = 8). Purification using Oasis HLB Cartridge provided the radiochemical purity of 99 ± 1%.

### 3.2. In Vitro Studies

**Figure 1:**
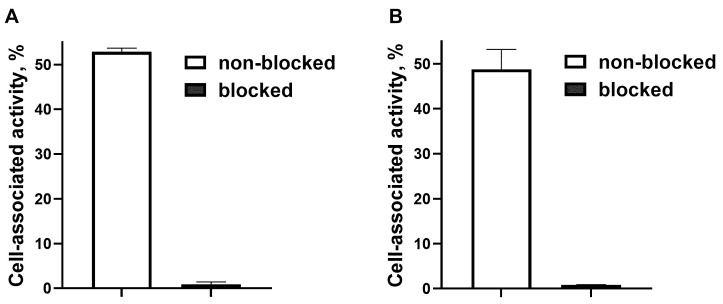
In vitro binding specificity of ^188^Re-ZHER2:41071 to HER2-expressing SKOV-3 (**A**) and SKBR-3 (**B**) cells. For pre-saturation of HER2, a 500-fold molar excess of non-radiolabeled polypeptide was added. The data are presented as an average (*n* = 3) value ± SD.

The results of in vitro binding specificity of ^188^Re-ZHER2:41071 are shown in Figure 1. Binding of ^188^Re-ZHER2:41071 to HER2-expressing SKOV-3 and SKBR-3 cells was significantly lower (*p* < 5 × 10^−5^) after saturating HER2 receptors in block groups, which indicates that the binding was HER2 mediated.

**Figure 2:**
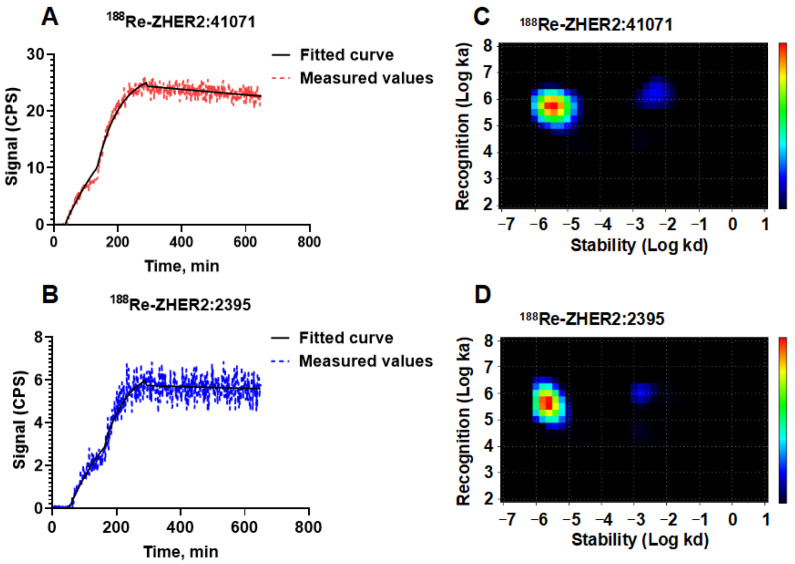
LigandTracer sensorgrams of ^188^Re-ZHER2:41071 (**A**) and ^188^Re-ZHER2:2395 (**B**) binding to HER2-expressing SKOV3 cells and results of InteractionMap analysis of binding of ^188^Re-ZHER2:41071 (**C**) and ^188^Re-ZHER2:2395 (**D**). Binding was measured at concentrations of 0.1 nM and 0.3 nM. Data are representatives from duplicates.

The affinities of ^188^Re-labeled ZHER2:41071 and ZHER2:2395 binding to HER2-expressing SKOV-3 cells were evaluated by InteractionMap analysis of the LigandTracer sensorgrams (Figure 2A,B). The InteractionMap analysis for both labeled Affibody^®^ molecules showed a one-to-two binding with high association and low dissociation rates, resulting in predominant affinity in low picomolar range (Figure 2C,D, Table 1).

**Figure 3:**
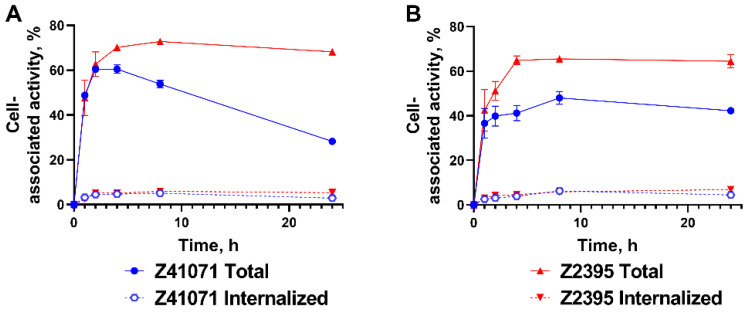
Binding and cellular processing of ^188^Re-ZHER2:41071 and ^188^Re-ZHER2:2395 by HER2-expressing SKOV-3 (**A**) and SKBR-3 (**B**) cells. Data are presented as an average (*n* = 3) value ± SD.

The results of cellular processing experiment of ^188^Re-ZHER2:41071 and ^188^Re-ZHER2:2395 in SKOV-3 and SKBR-3 cells is shown in Figure 3. Both ^188^Re-ZHER2:41071 and ^188^Re-ZHER2:2395 demonstrated a rapid binding to HER2-expressing cell during first 1 h of incubation. However, uptake and retention of the cell-associated activity was higher at later time points for ^188^Re-ZHER2:2395 having a residualizing label. Both variants showed slow internalization in SKOV-3 and SKBR-3 cells, less than 15% in 24 h.

### 3.3. In Vivo Studies

**Figure 4:**
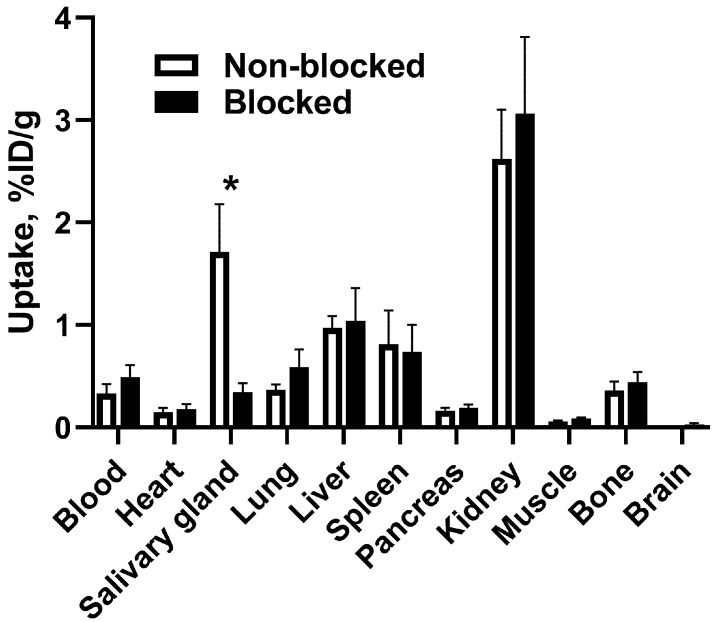
Effect of blocking of Na/I-symporter on biodistribution of ^188^Re-ZHER2:41071 in NMRI mice at 4 h p.i. The tissue uptake values were calculated as the percentage of injected dose per gram (%ID/g). Data are presented as an average (*n* = 4) value ± SD. Asterisk (*) indicate significant differences (*p* < 0.05) between blocked and non-blocked groups.

The effect of blocking of Na/I symporter on the biodistribution of ^188^Re-ZHER2:41071 in NMRI mice is shown in Figure 4. Blocking Na/I symporter by adding sodium iodide to drinking water significantly reduced (*p* < 0.005) the uptake of ^188^Re-ZHER2:41071 in salivary glands. In other normal organs, there was no significant difference between the uptakes in animals belonging to different groups.

**Figure 5:**
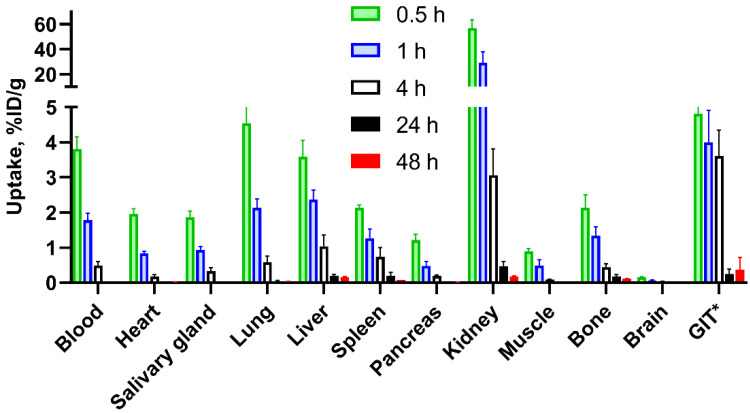
Biodistribution of ^188^Re-ZHER2:41071 in NMRI mice at 0.5, 1, 4, 24, and 48 h after injection. *GIT = gastrointestinal tract. Data are presented as %ID per whole sample. Data are presented as an average (*n* = 4) value ± SD.

Biodistribution of ^188^Re-ZHER2:41071 in NMRI mice is presented in Figure 5. The clearance of ^188^Re-ZHER2:41071 from blood was rapid and the clearance in other organs followed the blood clearance. The uptake of ^188^Re-ZHER2:41071 in gastrointestinal tract with content was less than 5% ID/g already at 0.5 h p.i. The initial uptake in kidney was the highest among all organs. However, very rapid decrease of kidneys-associated activity was observed. The activity in kidney was reduced from 57 ± 7%ID/g at 0.5 h p.i. to 3.1 ± 0.8%ID/g at 4 h p.i.

Estimated absorbed doses after injection of ^188^Re-ZHER2:41071 in humans are provided in Table 2. According to calculations using OLINDA/EXM 1.0, the highest absorbed doses are expected in kidneys, lower large intestine wall and heart wall. The expected effective dose is 0.0683 mSv/MBq.

**Figure 6:**
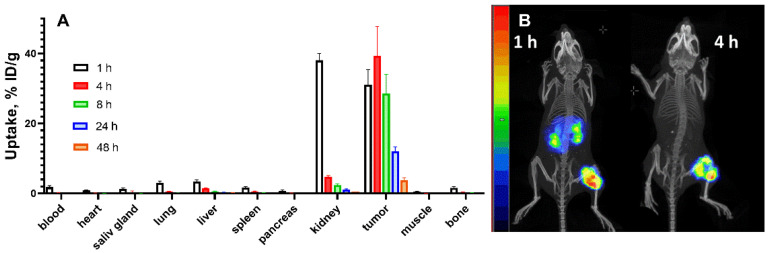
(**A**) Biodistribution of ^188^Re-ZHER2:41071 in BALB/C nu/nu mice bearing HER2-expressing SKOV-3 xenografts. Data are presented as an average (*n* = 4) value ± SD. (**B**) Imaging of BALB/C nu/nu mice bearing HER2-positive SKOV-3 xenografts using ^188^Re-ZHER2:41071 at 1 and 4 h after injection. The scale is linear showing arbitrary units normalized to a maximum count rate.

**Figure 7:**
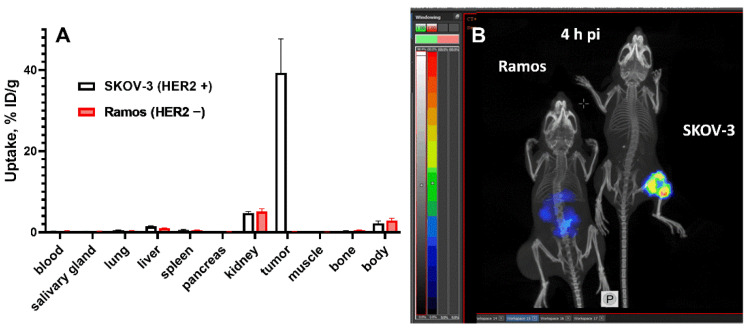
The HER2-specificity of ^188^Re-ZHER2:41071 accumulation in tumor xenografts was evaluated by comparison of the uptake in HER2-positive SKOV-3 and HER2-negative Ramos xenografts in BALB/C nu/nu mice 4 h after injection. (**A**) Biodistribution. Results of ex vivo measurements are presented as % ID/g ± SD (*n* = 4). (**B**) Imaging of ^188^Re-ZHER2:41071 in BALB/C nu/nu mice bearing SKOV-3 and Ramos xenografts 4 h after injection. The scale is linear showing arbitrary units normalized to a maximum count rate.

The data concerning the biodistribution of ^188^Re-ZHER2:41071 in BALB/C nu/nu mice bearing HER2-expressing SKOV-3 xenografts are presented in Figure 6 and Figure 7 and Table S2. In agreement with the biodistribution data in normal NMRI mice, ^188^Re-ZHER2:41071 cleared rapidly from blood and normal tissues including kidneys. The uptake in tumor was high, 31 ± 4%ID/g already 1 h after injection (Figure 6A and Table S2). By this time point, the tumor uptake was higher than the uptake in majority of organs and similar with the uptake in kidney. However, the tumor uptake remained to be high over time while the activity in kidneys declined rapidly. Already 4 h after injection, the tumor uptake was more than 8-fold higher than the renal uptake. This created a favorable precondition for the radionuclide therapy. Experimental imaging using microSPECT/CT confirmed the biodistribution data (Figure 6B).

The results from the in vivo specificity test showed that the uptake of ^188^Re-ZHER2:41071 in HER2-negative Ramos xenografts in BALB/C nu/nu mice was 245-fold (*p* < 0.0001) lower than that in HER2-expressing SKOV-3 xenografts, while the accumulation of radioactivity in other organs was the same (Figure 7A). The results were confirmed by the imaging performed 4 h after injection of ^188^Re-ZHER2:41071 both in SKOV-3 and Ramos xenografts (Figure 7B).

**Figure 8:**
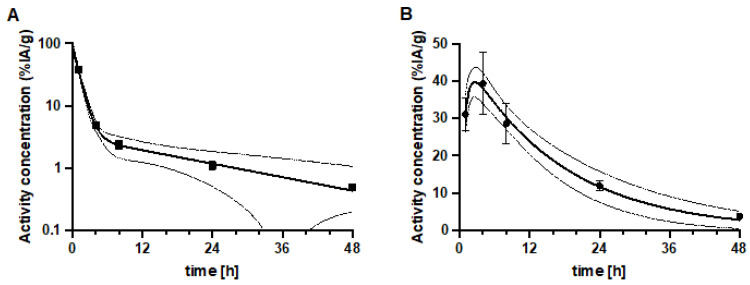
Time-activity concentration curves for (**A**) kidneys and (**B**) SKOV-3 tumor. The biodistribution data were fitted with bi-exponential curves; in the kidneys 90%/g with T_1/2_ = 44 min and 3%/g with T_1/2_ = 17 h and in the tumor uptake proceeded with T_1/2_ = 35 min to a maximum of 49%/g and clearance with T_1/2_ = 12 h. The fitted curves and its 95% confidence intervals are indicated.

An evaluation of dosimetry in mice suggested that the time-activity curves could be expressed as single exponential curves for all organs with R^2^ > 0.8. For the kidneys a bi-exponential curve was chosen to better account for the terminal clearance pattern in the kidneys (Figure 8A). Also, the tumor TAC could be better described by a bi-exponential curve (Figure 8B).

The absorbed dose values are indicated in Table 3. The kidneys receive the highest dose of the organs with physiological uptake at 341 mGy/MBq. The absorbed dose to the tumor is a factor 3 higher. The dose to the skeleton and bone marrow of 39 mGy/MBq might limit the total amount of activity that can be injected. In humans the maximum tolerated absorbed dose to the bone marrow is 2 Gy.

**Figure 9:**
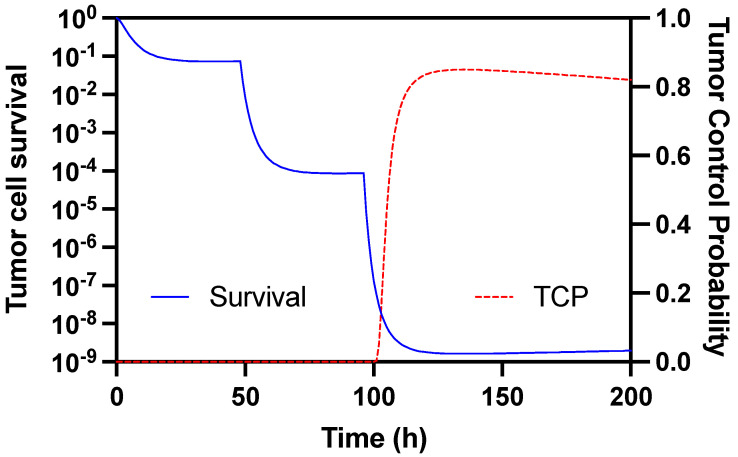
Prediction of SKOV-3 tumor cell survival and control probability after three cycles with 16 MBq ^188^Re-ZHER2:41071 administered at two-day intervals, based on the dosimetry and tumor radiation sensitivity.

A dosing scheme with three injections of 16 MBq given at two days interval leads to a tumor control probability (TCP) of 85% after 135 h or 5.6 days (Figure 9).

### 3.4. Experimental Radionuclide Therapy

**Figure 10:**
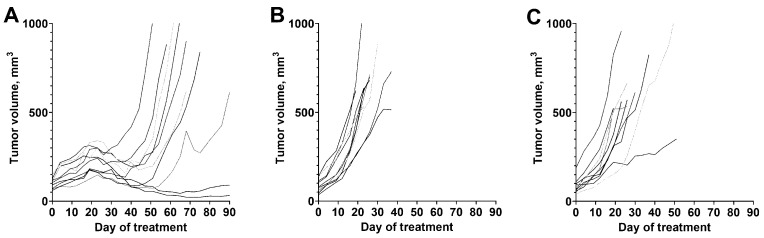
The growth of individual tumors in mice treated with (**A**) ^188^Re-ZHER2:41071 (three times with 5 µg, 16 MBq, dissolved in 10% ethanol in 0.9% saline), (**B**) non-labeled ZHER2:41071 (three times with 5 µg, dissolved in 10% ethanol in 0.9% saline), and (**C**) vehicle (three times with 10% ethanol in 0.9% saline).

**Figure 11:**
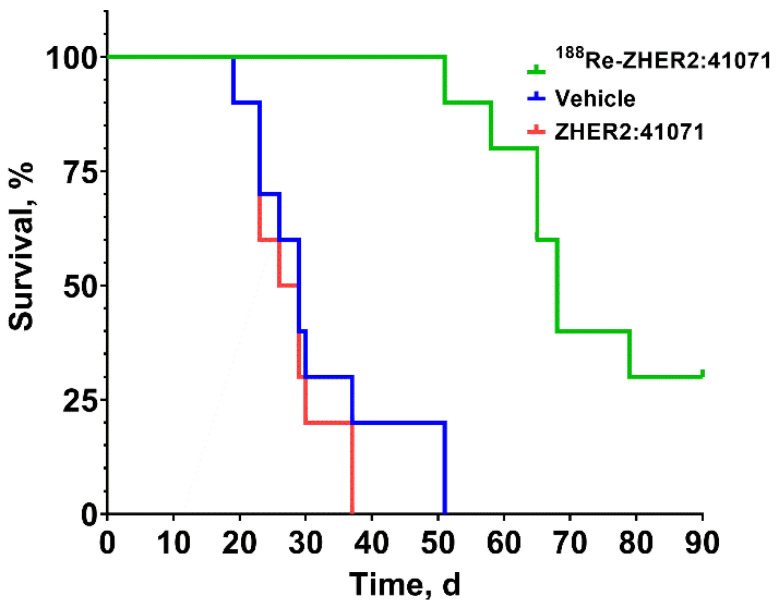
The survival of mice treated with ^188^Re-ZHER2:41071 (three times with 5 µg, 16 MBq, dissolved in 10% ethanol in 0.9% saline), non-labeled ZHER2:41071 (three times with 5 µg, dissolved in 10% ethanol in 0.9% saline), and vehicle (three times with 10% ethanol in 0.9% saline).

**Figure 12:**
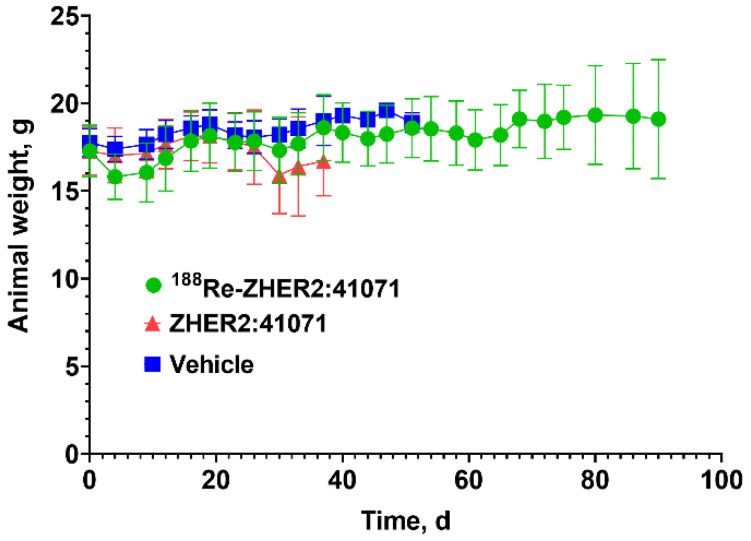
The average body weight of mice after treatment with^188^Re-ZHER2:41071 (three times with 5 µg, 16 MBq, dissolved in 10% ethanol in 0.9% saline), non-labeled ZHER2:41071 (three times with 5 µg, dissolved in 10% ethanol in 0.9% saline), and vehicle (three times with 10% ethanol in 0.9% saline).

The result of the experimental radionuclide therapy of xenografts are presented in Figure 10, Figure 11 and Figure 12. Figure 10 shows the growth of individual tumors in each group. The growth of tumors in control groups was rapid. The tumor volume doubling time was 9 ± 2 days in the group B (treated with non-labeled ZHER2:41071) and 12 ± 7 days in the group C (treated with vehicle). There was no significant difference (*p* > 0.05, unpaired *t*-test) between tumor doubling time in these groups, which suggests that the treatment with non-labeled ZHER2:41071 has no anti-tumor effect. All animals in the group B were sacrificed by day 37, either due reaching the tumor volume limit or due to tumors ulceration. In the group C, the last animal was sacrificed by day 51. The pattern of tumor growth in the group A treated with ^188^Re-ZHER2:41071 was different. A reduction of the tumor volume was observed starting from day 19 after treatment start. At this time point, the mean tumor volume was significantly smaller (*p* < 0.05, one-way ANOVA with Bonferroni correction for multiple comparison) than the volume in two other groups. After some delay, some tumors resumed growth. However, three animals were alive at the study termination (day 90).

The median survival in the treatment group (A) was 68 days, which was significantly longer (*p* < 0.0001 in the log-rank Mantel-Cox test) than in the control groups (B, 27.5 days; C, 29 days) (Figure 11). There was no difference in survival of mice treated with non-labeled ZHER2:41071 (group B) or vehicle (group C) (*p* > 0.05 in the log-rank Mantel-Cox test).

The therapy was well tolerated. Behavior of mice in the treatment group and their appearance did not differ from behavior and appearance of animals in control groups. The average animal weight did not differ significantly between the treated group and the control groups (Figure 12). According to the pathology examination “in the examined material there were no lesions found in liver or kidney that suggest toxicity of the treatment regimes” (Figures S2 and S3).

**Figure 13:**
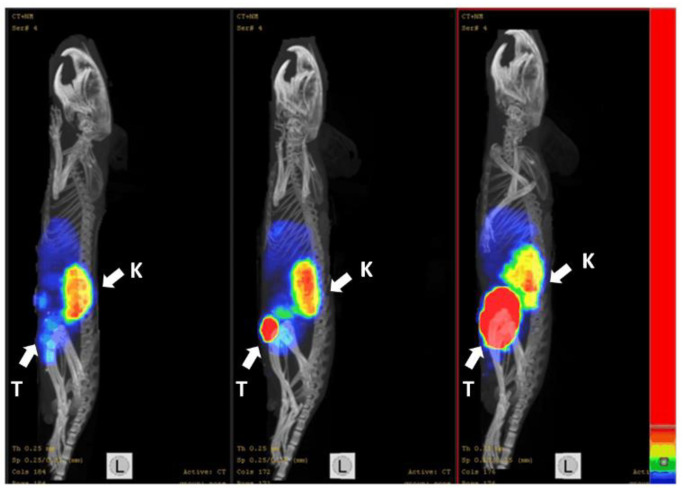
The SPECT-CT imaging using ^99m^Tc-ZHER2:41071 of mice with different tumor remission level after treatment with ^188^Re-ZHER2:41071. Imaging was performed at day 35 after treatment start. The scale is linear showing arbitrary units normalized to a count rate in kidneys. The arrows with the letter “T” point to the tumors, and the arrows with the letter “K” point to the kidneys.

Radionuclide molecular imaging of mice using ^99m^Tc-ZHER2:41071 during the treatment (Figure 13) enabled visualization of HER2-expressing xenografts. The signal from tumors reflected their size and correlated with the outcome of the therapy.

## 4. Discussion

Affibody^®^ molecules have shown an efficient targeting of cancer-related molecular abnormalities [10]. The Affibody molecule ZHER2:V2 with –GGGC chelator on C-terminus has demonstrated a combination of an efficient tumor targeting with low renal retention of activity when labeled with ^99m^Tc [19] or ^188^Re [31]. However, a deep re-engineering of the scaffold [20,21] resulted not only in improved yield of peptide synthesis, lower binding to blood proteins and lower immunogenic potential. Essentially, a new targeting protein, ZHER2:41071, has been created, and its further clinical translation required full preclinical re-evaluation. This study proved that ZHER2:41071 can be efficiently labeled with ^188^Re both in a small scale and in a large scale, which is required in clinics. A purification using solid-phase extraction provided a high purity (Figure S1) necessary for clinical translation. The labeled protein has shown a clearly specific binding to two HER2 expressing cell lines (Figure 1). To evaluate effect of composition of a chelator on cellular processing, we used ^188^Re-labeled ZHER2:2395 Affibody molecule for comparison. ZHER2:2395 has the same binding site as ZHER2:41071, but it is framed into the first generation Affibody scaffold. Earlier studies [32] demonstrated strong residualizing character of ^188^Re-KVDC complex, which was manifested by a strong cellular retention in vitro and by a strong retention of activity in kidneys in vivo. Thus, the difference between cellular retention of ^188^Re-ZHER2:41071 and ^188^Re-ZHER2:2395 should be determined by the difference of residualizing properties of ^188^Re-KVDC and ^188^Re-GGGC complexes. Affinity measurements (Figure 2; Table 1) showed very similar kinetics of binding of ^188^Re-ZHER2:41071 and ^188^Re-ZHER2:2395 to living HER2-expressing cells. Particularly, the ^188^Re-ZHER2:41071 binding was characterized by two types of interaction, which is typical for HER2 binders and is most likely caused by difference of binding to non-dimerized and dimerized HER2 [33]. Importantly, the most abundant interaction with the affinity of 5 ± 3 pM ensures a stable tumor retention of ^188^Re-ZHER2:41071. A comparison of binding and cellular processing of ^188^Re-ZHER2:41071 and ^188^Re-ZHER2:2395 in vitro demonstrated lower uptake and retention of ^188^Re-ZHER2:41071 at late time point, which suggested an effect of a non-residualizing label.

Our previous data indicated high in vivo stability of the technetium label in ^99m^Tc-ZHER2:41071 [21]. However, rhenium has a greater tendency to re-oxidation in vivo compared to technetium, which might result in a release of radioperrhenate [23]. The radioperrhenate is taken up by a saturable mechanism in tissues expressing Na/I symporter, such as thyroid, stomach and salivary gland [34]. Our experiment in normal mice (Figure 4) demonstrated that a pre-saturation of Na/I symporters by cold iodide significantly (*p* < 0.005) reduced uptake of ^188^Re in salivary gland after injection of ^188^Re-ZHER2:41071. This indicated that there might be a formation of radioperrhenate during metabolism of ^188^Re-ZHER2:41071. Such feature could lead to undesirable elevated doses to a number of tissues. However, the saturable character of radioperrhenate uptake enables its prevention by pre-administration of a stable iodine or sodium perchlorate, similarly to procedures that are recommended for radionuclide therapy using ^131^I-MIBG [35]. Our further in vivo experiments included in vivo blocking of Na/I symporters by cold iodide. Further experiments revealed a rapid clearance of activity from all tissues after injection of ^188^Re-ZHER2:41071 (Figure 5). Importantly, a substantial initial uptake in kidney was followed by a rapid release of activity. An upscaling of the murine data for humans indicated that such pattern of biodistribution would result in quite favorable equivalent dose of 0.0683 mSv/MBq (Table 2).

The biodistribution measurements in tumor-bearing mice (Figure 6) confirmed the finding in normal mice. The tumor uptake exceeded renal uptake after one hour post injection and remained appreciably higher at later time points. The uptake in HER2-positive SKOV-3 xenografts was much higher (*p* < 0.0001) than in HER2-negative Ramos xenografts (Figure 7). This indicates that the in vivo accumulation of ^188^Re-ZHER2:41071 in tumor xenografts is highly HER2-specific.

A tumor response to irradiation depends on absorbed dose and its dose rate. The external beam therapy is typically provided in daily 2 Gy fractions at high dose rate, and a curative effect is expected at the absorbed dose to tumor of 60 Gy [36]. The fractionation permits a re-oxygenation of radioresistant hypoxic cells making them more radiosensitive and repair of DNA damage predominantly in normal tissue. On the opposite, a targeted radionuclide therapy is associated with a low dose rate and is typically given in cycles separated by several weeks. Such treatment pattern results in much lower efficacy. For example, the largest anti-tumor effect (tumor reduction by 57%) of treatment of pancreatic neuroendocrine tumors with ^177^Lu-DOTATATE was obtained after a total absorbed dose of 170 Gy [37]. Due to relatively short half-life of ^188^Re the dose rate per administered activity would be higher comparing with the dose rate provided by the most commonly used therapeutic radionuclide ^177^Lu (T_1/2_ = 6.73 d), which is favorable for the therapy outcome. Moreover, a ^188^W/^188^Re generator might be placed at a hospital providing elution of ^188^Re every 48 h. This would provide a fractionated treatment regimen, further increasing the efficacy of the therapy.

In this study, we have applied for selection of treatment regimen a tumor control probability model, which demonstrated an efficient prediction of an experimental therapy outcome in several of our previous studies [38,39,40]. This modelling suggested that three injections of 16 MBq ^188^Re-ZHER2:41071 given 48 h apart would provide a substantial tumor probability control (Figure 9). This prediction was confirmed in an experimental preclinical therapy (Figure 10 and Figure 11). We observed a significant delay of tumor growth in mice treated with three injections of 16 MBq ^188^Re-ZHER2:41071 (median survival 68 days) compared with animals treated with vehicle (median survival 29 days). The anti-tumor effect was caused by irradiation and not by an anti-tumor effect of ZHER2:41071 Affibody molecules, since treatment with non-labeled Affibody molecule did not cause any delay in the tumor growth (median survival 27.5 days) compared with animals treated with vehicle. The treatment had no visible effect on animal health or weight. The histopathology did not reveal any pathological changes in kidneys, which received the highest absorbed dose (3 × 5.4 Gy) among normal organs.

In advanced settings, targeted radionuclide therapy of neuroendocrine tumors using ^177^Lu-DOTATATE includes one or several imaging of patients after every injection [41]. This enables both performing patient-specific dosimetry and estimation a response of each patient to the treatment, making the therapy personalized. Such therapy response monitoring is impossible within the proposed scheme when injections of ^188^Re-ZHER2:41071 are given within one week. However, ^99m^Tc-ZHER2:41071 might act as a theranostic counterpart of ^188^Re-ZHER2:41071. The estimated effective dose for ^99m^Tc-ZHER2:41071 should be only 0.00066 mSv/MBq [21], which would permit multiple imaging procedures. Our imaging studies (Figure 13) suggested that this would enable discrimination between responding and non-responding patients.

## 5. Conclusions

^188^Re-ZHER2:41071 provides high specific accumulation of activity in human HER2-expressing xenografts and low renal retention of activity in a murine model.

The experimental radionuclide therapy using ^188^Re-ZHER2:41071 enables enhancement of survival of treated mice with human tumors without toxicity to the critical organ (i.e., the kidneys).

## Figures and Tables

**Table 1:** Apparent kinetic parameters of ^188^Re-ZHER2:41071 interaction with HER2-expressing SKOV-3 cells.

Interaction	k_a_ (M^−1^s^−1^)	k_d_ (s^−1^)	K_D_ (M)	Weight (%)
^188^Re-ZHER2:41071				
1	[5.7 ± 0.4] × 10^5^	[2.6 ± 1.3] × 10^−6^	[5 ± 3] × 10^−12^	76
2	[1.3 ± 0.3] × 10^6^	[5.5 ± 3.5] × 10^−4^	[5 ± 4] × 10^−9^	6
^188^Re-ZHER2:2395				
1	[5.4 ± 2.0] × 10^5^	[3.5 ± 1.5] × 10^−6^	[7 ± 4] × 10^−12^	75
2	[7.7 ± 6.5] × 10^5^	[2.5 ± 1.5] × 10^−3^	[27 ± 18] × 10^−9^	8

**Table 2:** Calculated absorbed dose (mGy/MBq) for ^188^Re-ZHER2:41071 in humans using OLINDA/EXM 1.0.

Organ	Absorbed Dose (mGy/MBq)	Organ	Absorbed Dose (mGy/MBq)
Adrenals	0.007	Muscle	0.005
Brain	0.001	Ovaries	0.007
Breasts	0.007	Pancreas	0.010
Gallbladder Wall	0.007	Red Marrow	0.012
Lower Large Intestines Wall	0.227	Osteogenic Cells	0.062
Small Intestine	0.044	Skin	0.006
Stomach Wall	0.208	Spleen	0.020
Upper Large Intestines Wall	0.007	Thymus	0.007
Heart Wall	0.131	Thyroid	0.007
Kidneys	0.244	Urinary Bladder Wall	0.007
Liver	0.029	Uterus	0.007
Lungs	0.031	Total Body	0.015
Effective Dose	0.0683 (mSv/MBq)		

**Table 3:** Dosimetry results for ^188^Re-ZHER2:41071 in NMRI mice with Time Integrated Activity Concentration Coefficients, the absorbed per injected activity, per 16 MBq therapy cycle and cumulative absorbed dose after 3 × 16 MBq.

Organ	TIACC (h/g)	Absorbed Dose per IA (mGy/MBq)	Absorbed Dose per 16 MBq (Gy)	Absorbed Dose after 3 Cycles of 16 MBq (Gy)
Brain	0.0024	8	0.1	0.4
Large Intestines	0.0318	31	0.5	1.5
Small Intestine	0.0443	29	0.5	1.4
Stomach Wall	0.0329	40	0.6	1.9
Heart	0.0296	29	0.5	1.4
Kidneys	1.3013	341	5.4	16.3
Liver	0.1835	69	1.1	3.3
Lungs	0.0997	34	0.5	1.6
Pancreas	0.0234	43	0.7	2.1
Skeleton	0.0823	39	0.6	1.9
Spleen	0.0863	40	0.6	1.9
Thyroid	0.0457	16	0.3	0.8
Total Body	0.0157	34	0.5	1.6
Tumor (100 mg)	4.47	1014	16.2	48.7

## Data Availability

Data is contained within the article or supplementary material.

## Supplementary Materials

The following supporting information can be downloaded at: https://www.mdpi.com/article/10.3390/pharmaceutics14051092/s1, Figure S1: RadioHPLC chromatogram of ^188^Re-ZHER2:41071; Table S1: Biodistribution of ^188^Re-ZHER2:41071 in normal NMRI mice; Table S2: Biodistribution of ^188^Re-ZHER2:41071 in BALB/C nu/nu mice bearing HER2-expressing SKOV-3 xenografts; Figure S2: Results of pathological examination of kidneys. HE, 100× and 400× of kidney in mouse from ^188^Re-ZHER2:41071 group (**A**,**B**), vehicle group (**C**,**D**) and ZHER2:41071 group (**E**,**F**); Figure S3: Results of pathological examination of livers. HE, 100× and 400× of liver in mouse from ^188^Re-ZHER2:41071 group (**A**,**B**), vehicle group (**C**,**D**) and ZHER2:41071 group (**E**,**F**). Arrows indicate a small and a large nucleus.
