# Supplementary material for: Experimental Therapy of HER2-Expressing Xenografts Using the Second-Generation HER2-Targeting Affibody Molecule 188Re-ZHER2:41071
Source: Pharmaceutics. 2022 May 20;14(5):1092. doi: 10.3390/pharmaceutics14051092 (PMC9146794; doi:10.3390/pharmaceutics14051092)
Supplement: Supplementary file 1 [file pharmaceutics-14-01092-s001.zip › pharmaceutics-1728360-supplementary.pdf]

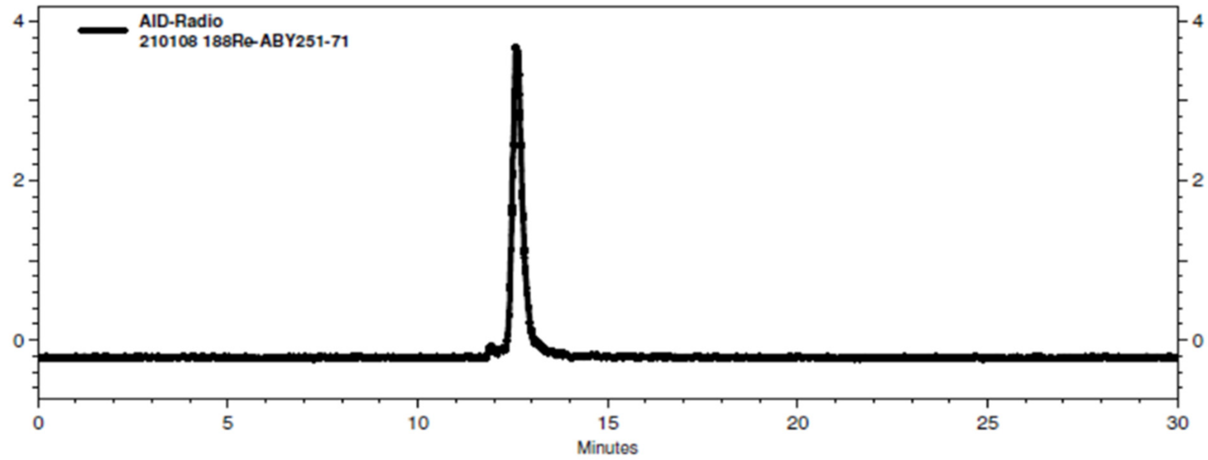

Figure S1. RadioHPLC chromatogram of ^{188}Re-ZHER2:41071.

Table S1. Biodistribution of ^{188}Re-ZHER2:41071 in normal NMRI mice.

	Uptake, %ID/g				
	0.5 h	1 h	4 h	24 h	48 h
blood	3.8±0.3	1.8±0.2	0.5±0.1	0.01±0.00	0.009±0.004
heart	1.9±0.2	0.84±0.05	0.18±0.05	0.01±0.01	
salivary gland	1.9±0.2	0.9±0.1	0.34±0.09	0.01±0.00	
lung	4.5±0.7	2.1±0.3	0.6±0.2	0.05±0.02	
liver	3.6±0.5	2.4±0.3	1.0±0.3	0.20±0.04	0.15±0.03
spleen	2.1±0.1	1.3±0.3	0.7±0.3	0.2±0.1	0.080±0.004
pancreas	1.2±0.2	0.5±0.1	0.19±0.03	0.010±0.004	
kidney	57±7	29±9	3.1±0.8	0.5±0.1	0.17±0.02
muscle	0.9±0.1	0.5±0.2	0.09±0.01	0.005±0.001	0.004±0.001
bone	2.1±0.4	1.3±0.2	0.4±0.1	0.17±0.06	0.106±0.006
brain	0.15±0.02	0.07±0.02	0.03±0.01	0.003±0.001	
stomach*	1.6±0.2	1.7±0.4	1.9±0.3	0.04±0.02	0.04±0.03
small intestine*	2.2±0.5	1.8±0.4	0.5±0.2	0.05±0.02	0.04±0.04
large intestine*	0.7±0.1	0.3±0.1	0.5±0.5	0.07±0.04	0.1±0.1
caecum*	0.37±0.05	0.21±0.03	0.7±0.2	0.10±0.06	0.1±0.1
carcass*	26±2	14±1	3.4±0.7	0.4±0.1	0.26±0.02

* Data for stomach, small intestine, large intestine, and caecum are presented as %ID per whole sample with content. Data for carcass are presented for whole sample. The data are presented as average (n=4) values ± SD.

Table S2. Biodistribution of ^{188}Re-ZHER2:41071 in BALB/C nu/nu mice bearing HER2-expressing SKOV-3 xenografts.

	Uptake, %ID/g				
	1h	4 h	8h	24 h	48 h
Blood	1.8±0.5	0.23 ±0.07	0.036±0.003	0.023±0.009	0.005±0.005
heart	0.9±0.1	0.12 ±0.01	0.03 ±0.01	0.024±0.008	0.02±0.02
salivary gland	1.3±0.3	0.4±0.4	0.04 ±0.01	0.025±0.007	0.03±0.01
lung	3.0±0.6	0.5±0.1	0.13 ±0.02	0.079±0.007	0.07±0.03
liver	3.4±0.5	1.4±0.1	0.6±0.1	0.31±0.04	0.23±0.04
spleen	1.7±0.2	0.6±0.1	0.23 ±0.03	0.17±0.03	0.12±0.01
pancreas	0.7±0.3	0.11 ±0.04	0.02 ±0.01	0.017±0.001	0.015±0.006
kidney	38±2	4.8±0.4	2.4±0.4	1.1±0.2	0.5±0.1
tumor	31±4	39±8	29±6	12±1	3.8±0.6
muscle	0.5±0.1	0.06 ±0.02	0.02 ±0.01	0.016±0.008	0.006±0.002
bone	1.6±0.4	0.34 ±0.04	0.20 ±0.04	0.18±0.03	0.18±0.06
brain	0.07 ±0.02	0.015±0.004	0.01 ±0.01	0.003±0.001	0.004
stomach*	0.8±0.2	0.3±0.2	0.02 ±0.01	0.05±0.04	0.0
small intestine*	1.2±0.3	0.3±0.2	0.07 ±0.01	0.034±0.005	0.018±0.007
large intestine*	0.3±0.1	0.26 ±0.06	0.09 ±0.04	0.2±0.2	0.04±0.02
caecum*	0.2±0.1	0.6±0.1	0.19 ±0.05	0.3±0.3	0.06±0.04
carcass*	12±2	2.2±0.6	0.60 ±0.02	0.36±0.02	0.25±0.04

* Data for stomach, small intestine, large intestine, and caecum are presented as %ID per whole sample with content. Data for carcass are presented as %ID for whole sample. The data are presented as average (n=4) values ± SD.

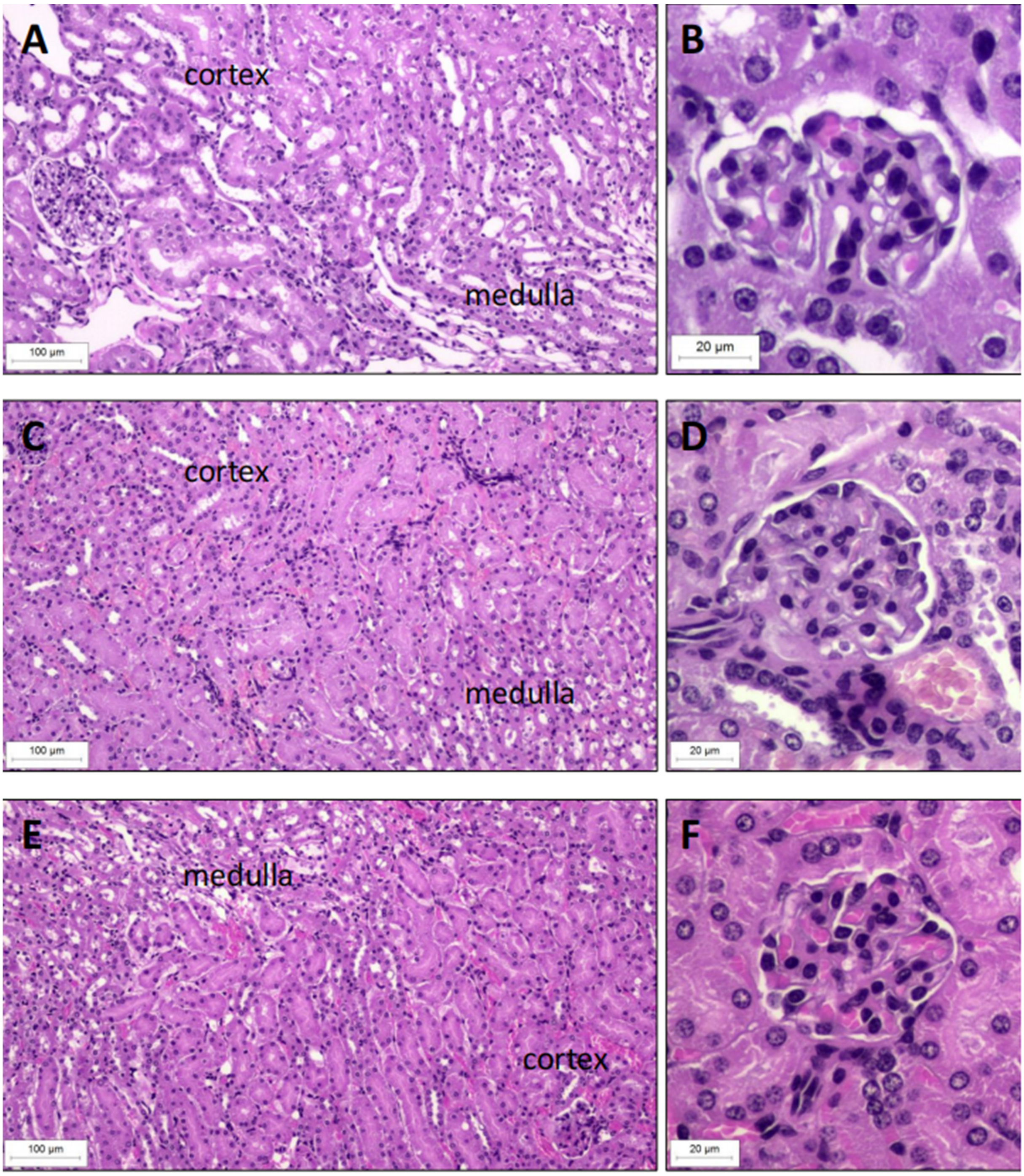

Figure S2. Results of pathological examination of kidneys. HE, 100x and 400x of kidney in mouse from ^{188}Re-ZHER2:41071 group (**A**,**B**), vehicle group (**C**,**D**) and ZHER2:41071 group (**E**,**F**).

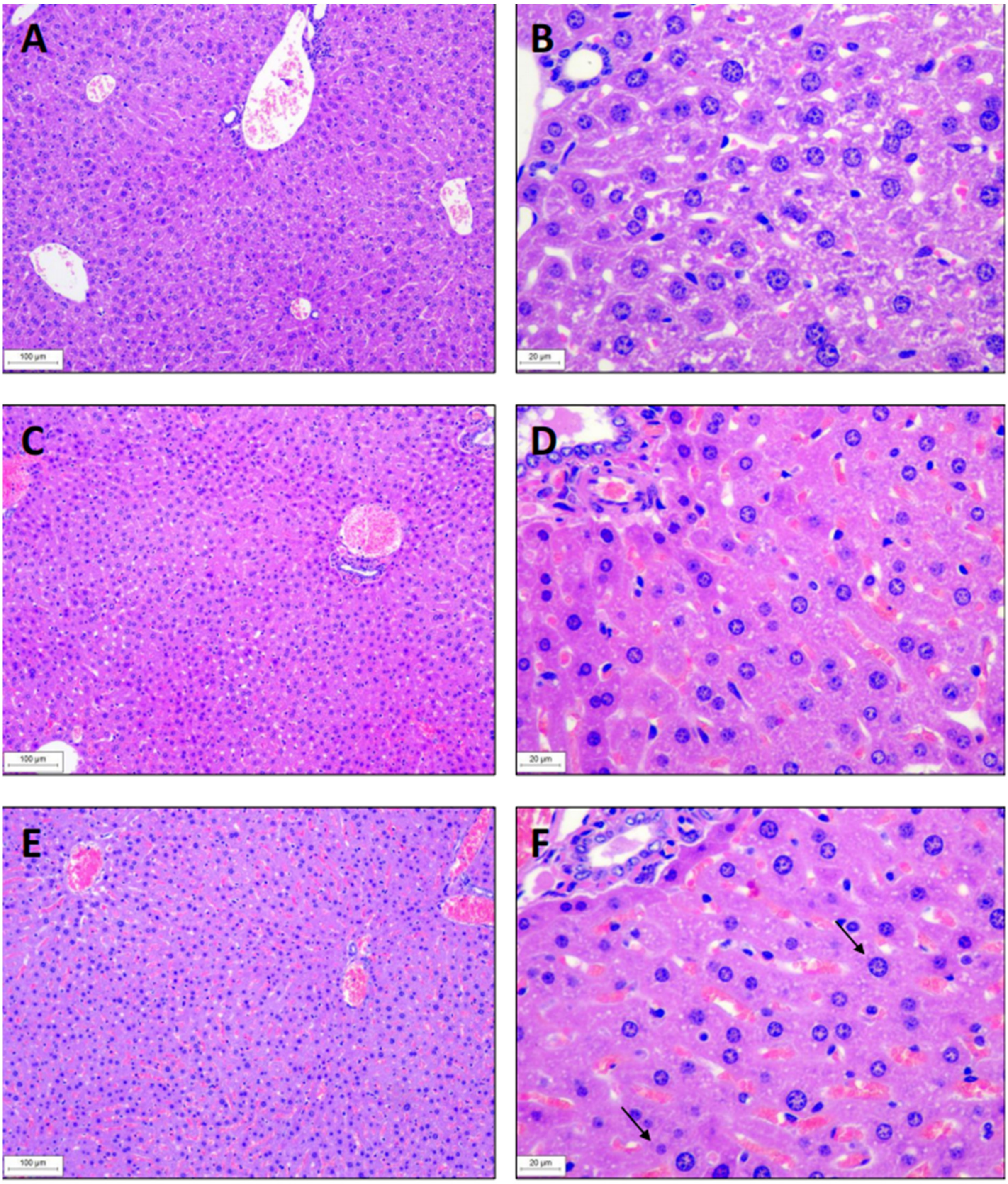

Figure S3. Results of pathological examination of livers. HE, 100x and 400x of liver in mouse from ^{188}Re-ZHER2:41071 group (**A**,**B**), vehicle group (**C**,**D**) and ZHER2:41071 group (**E**,**F**). Arrows indicate a small and a large nucleus.
